# Supplementary material for: Thermally Remendable Polyurethane Network Cross-Linked via Reversible Diels–Alder Reaction
Source: Polymers (Basel). 2021 Jun 10;13(12):1935. doi: 10.3390/polym13121935 (PMC8230680; doi:10.3390/polym13121935)
Supplement: Supplementary file 1 [file polymers-13-01935-s001.zip › polymers-1242193-supplementary.pdf]

polymers

Supplementary material: Thermally Remendable Polyurethane Network Cross-Linked via Reversible Diels-Alder Reaction

Elena Platonova, Islam Chechenov, Alexander Pavlov, Vitaliy Solodilov, Egor Afanas'ev, Alexey Shapagin, and Alexander Polezhaev

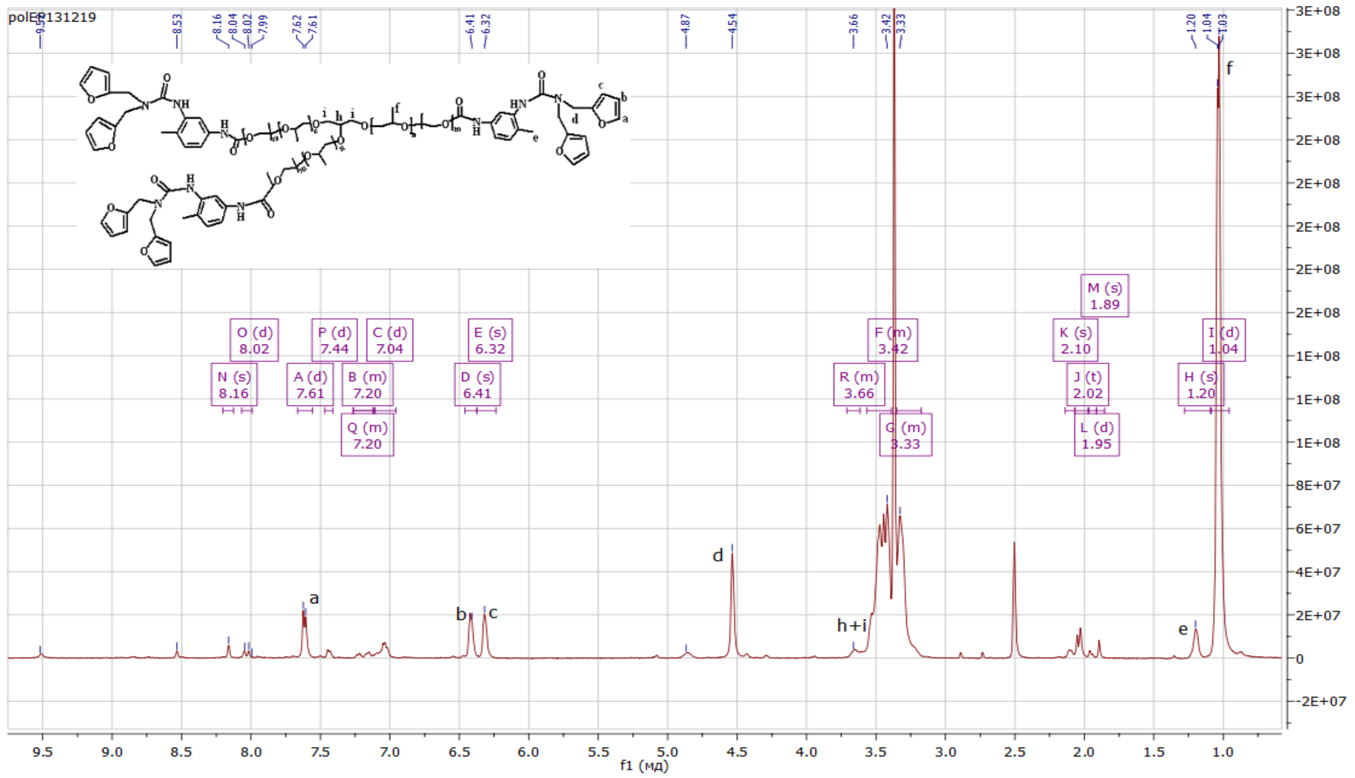

Figure S1. ^{1}H NMR spectrum of PU-V0 in DMSO-d_6.

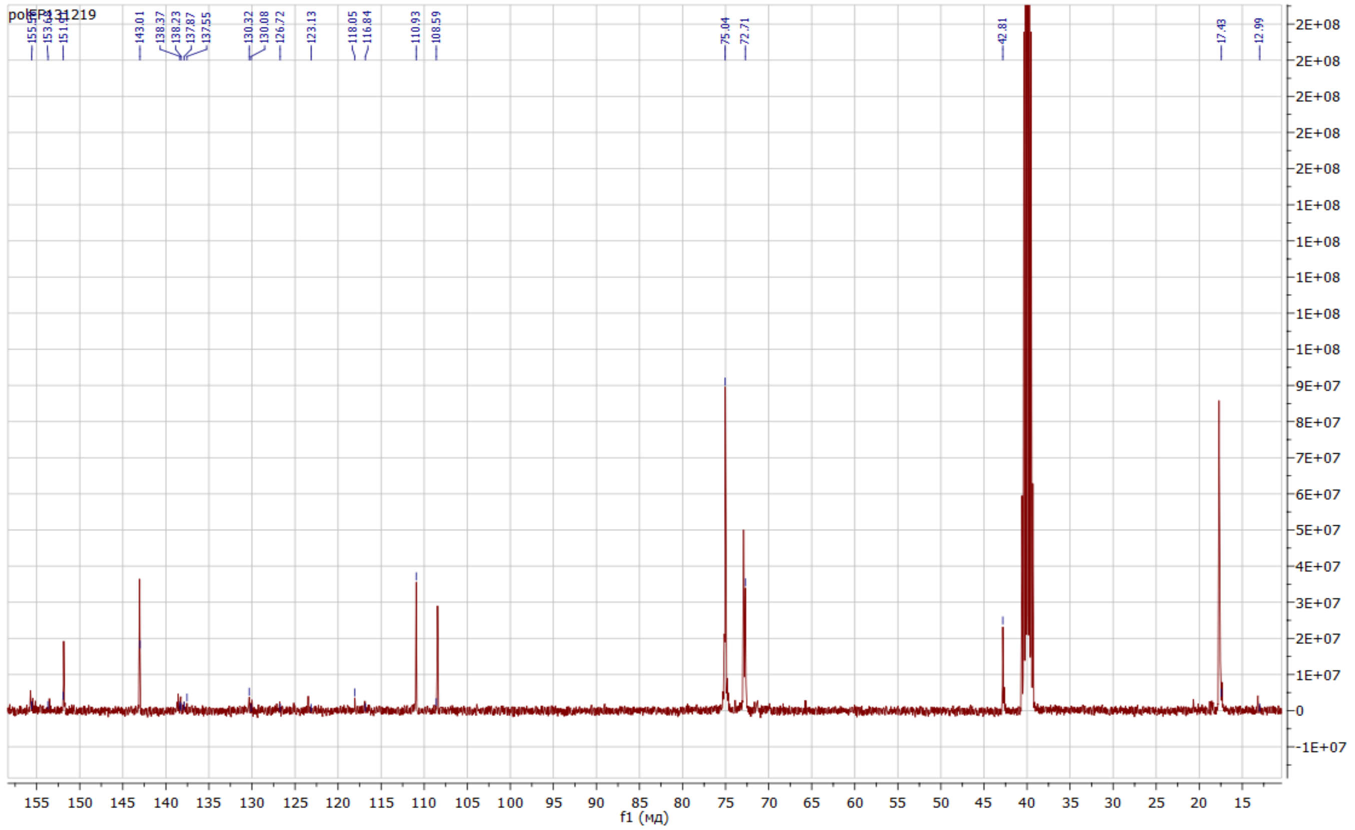

Figure S2. ^{13}C NMR spectrum of PU-V0 in DMSO-d_6.

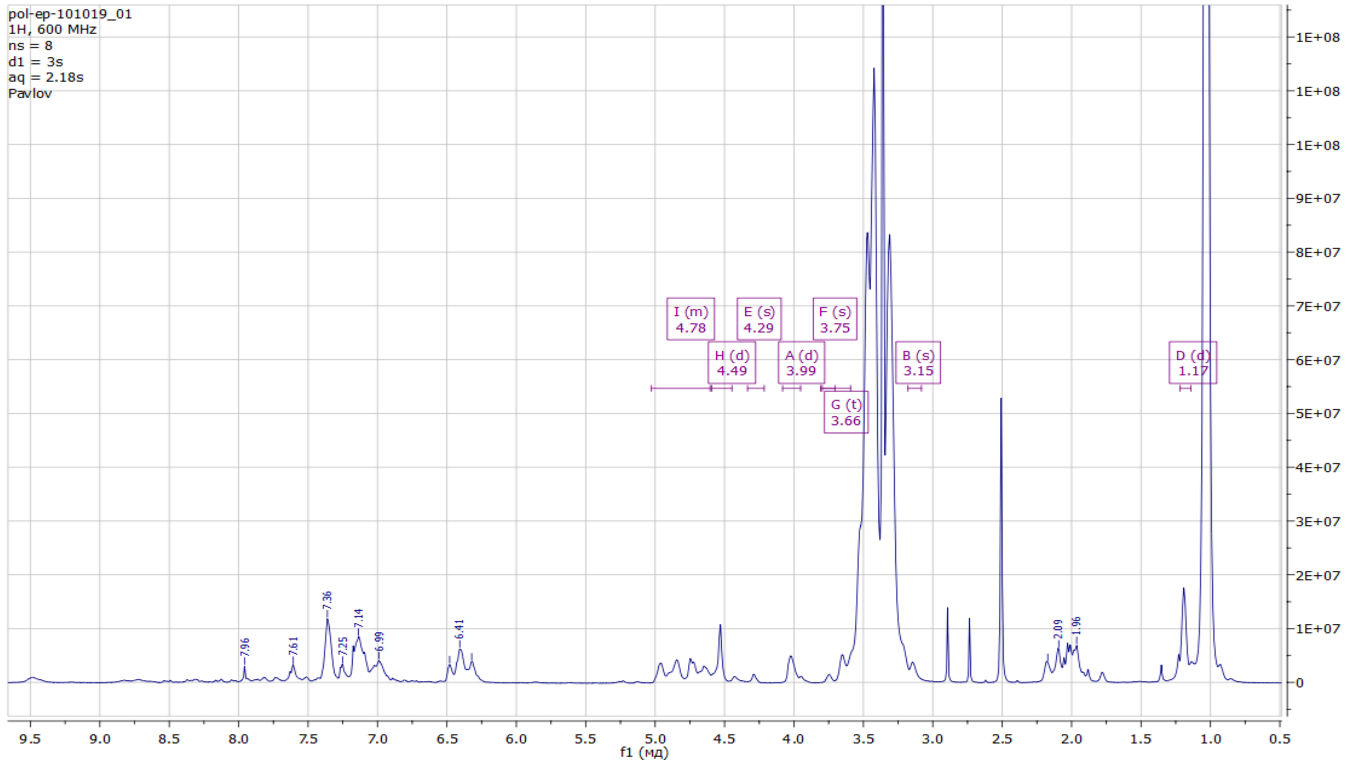

Figure S3. ^{1}H NMR spectrum of PU-V1.0 in DMSO-d_6.

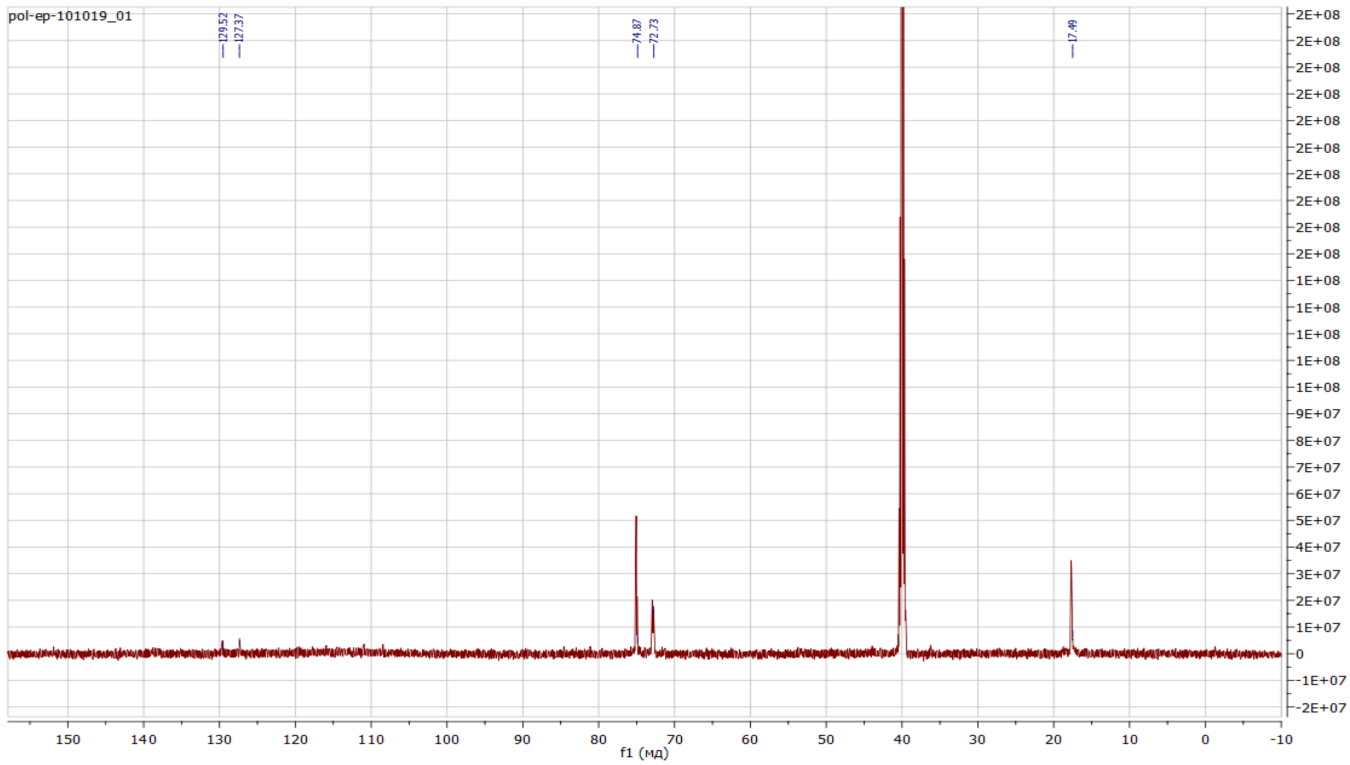

Figure S4. ^{13}C NMR spectrum of PU-V1.0 in DMSO-d_6.

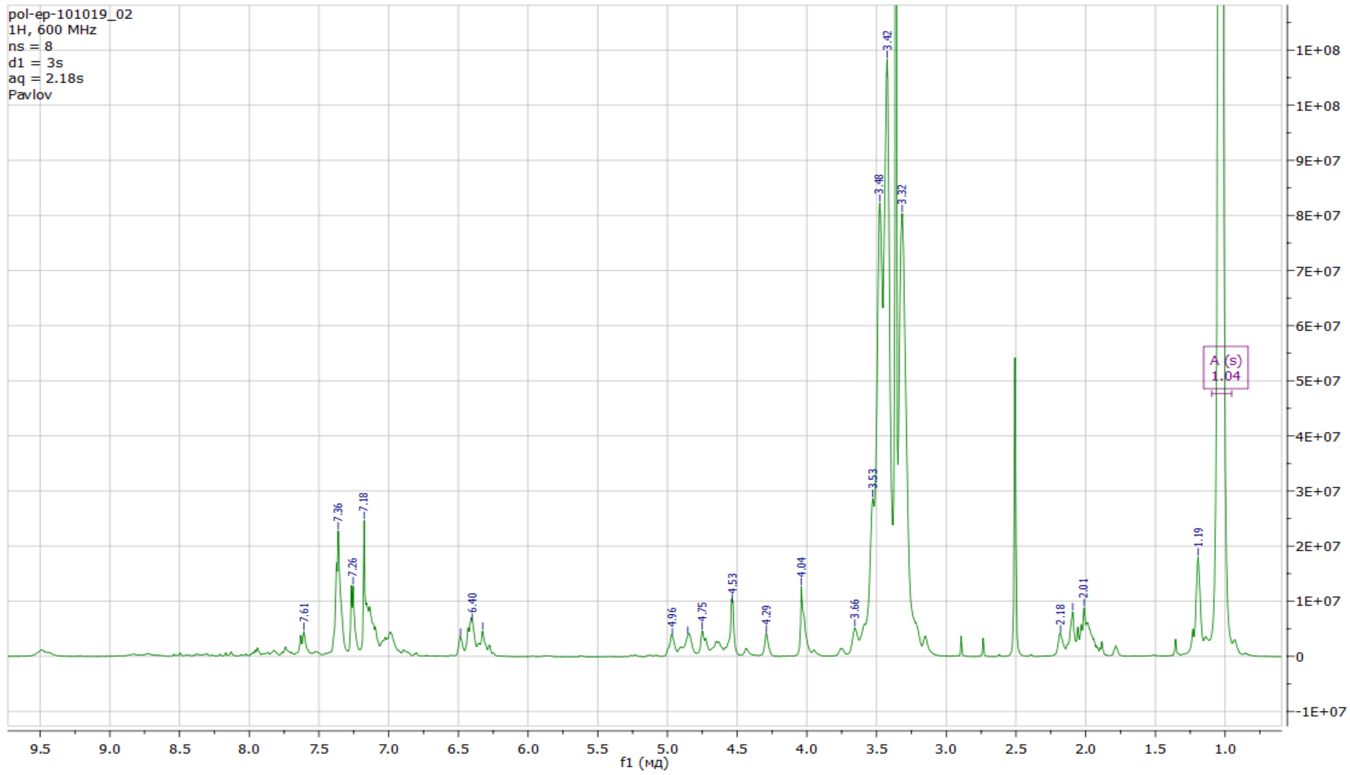

Figure S5. ^{1}H NMR spectrum of PU-V2.0 in DMSO-d_6.

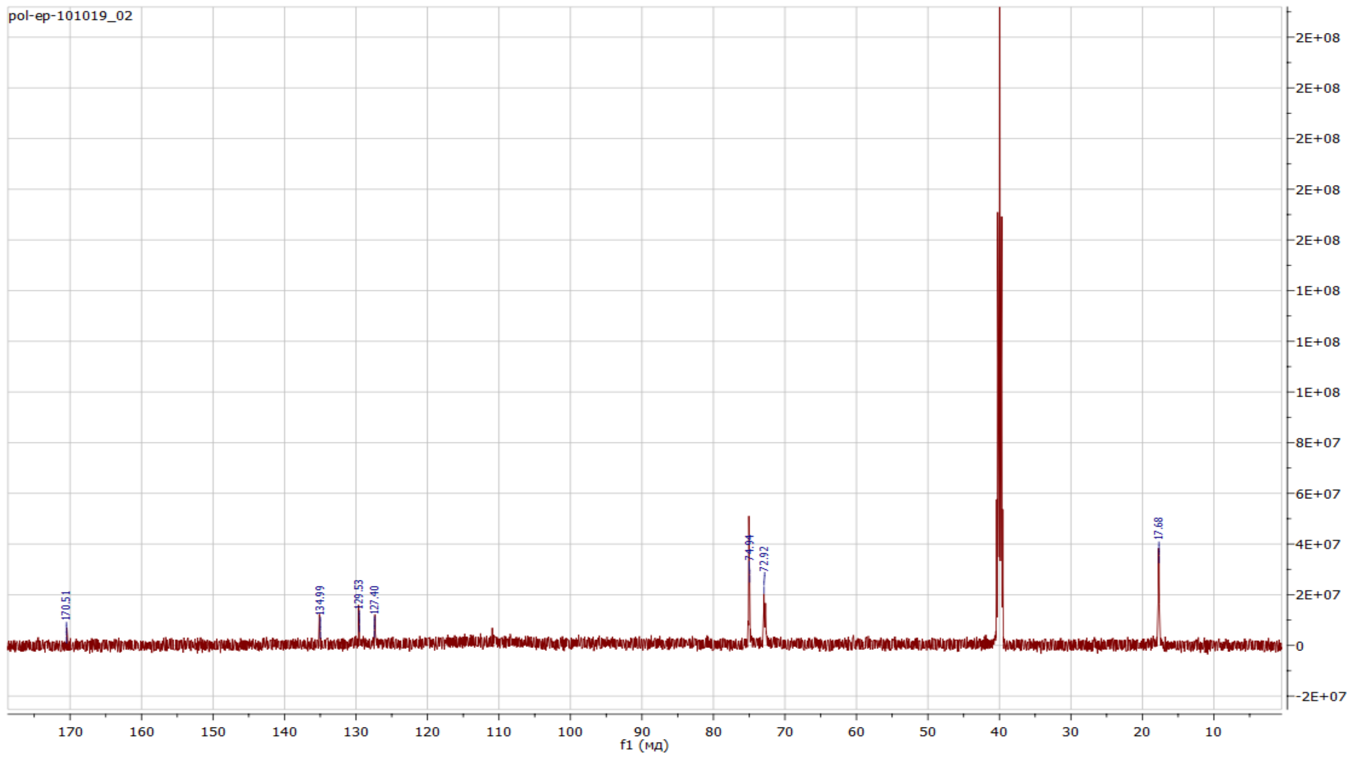

Figure S6. ^{13}C NMR spectrum of PU-V2.0 in DMSO-d6.

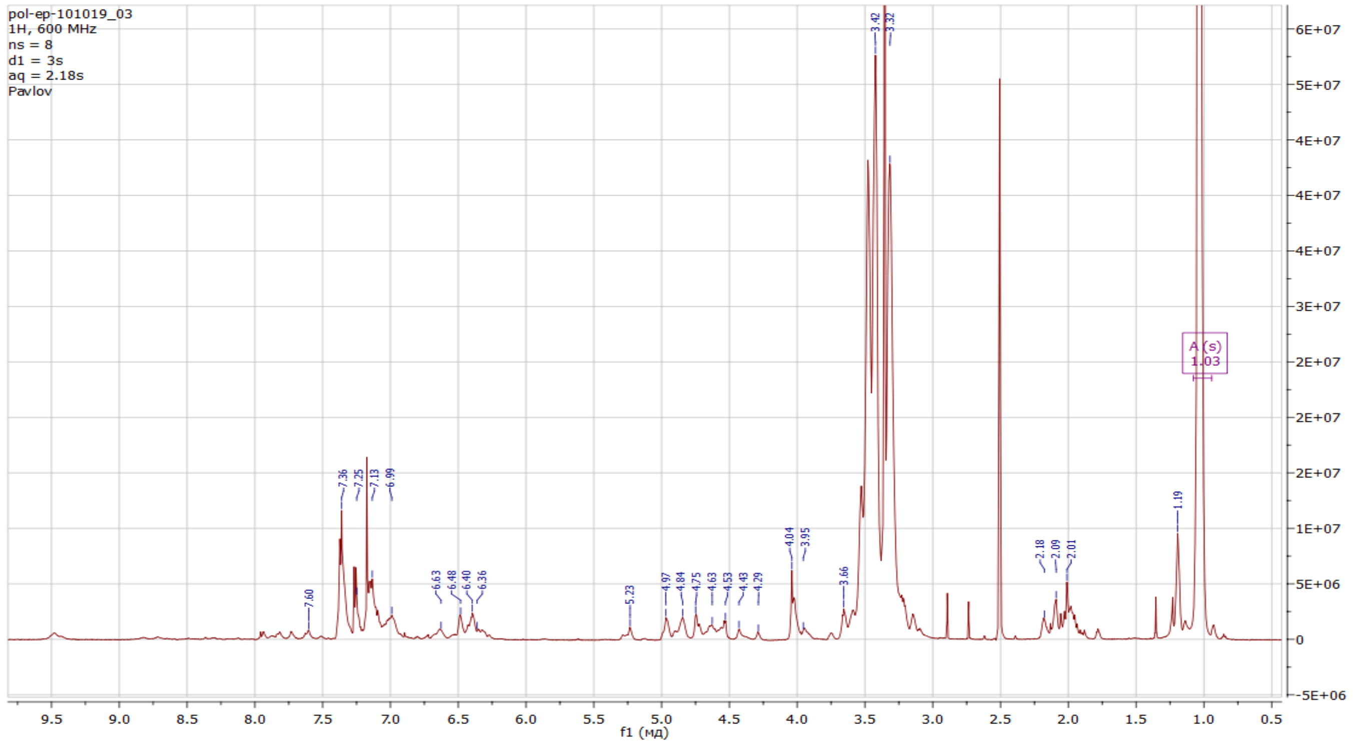

Figure S7. ^{1}H NMR spectrum of PU-V3.0 in DMSO-d6.

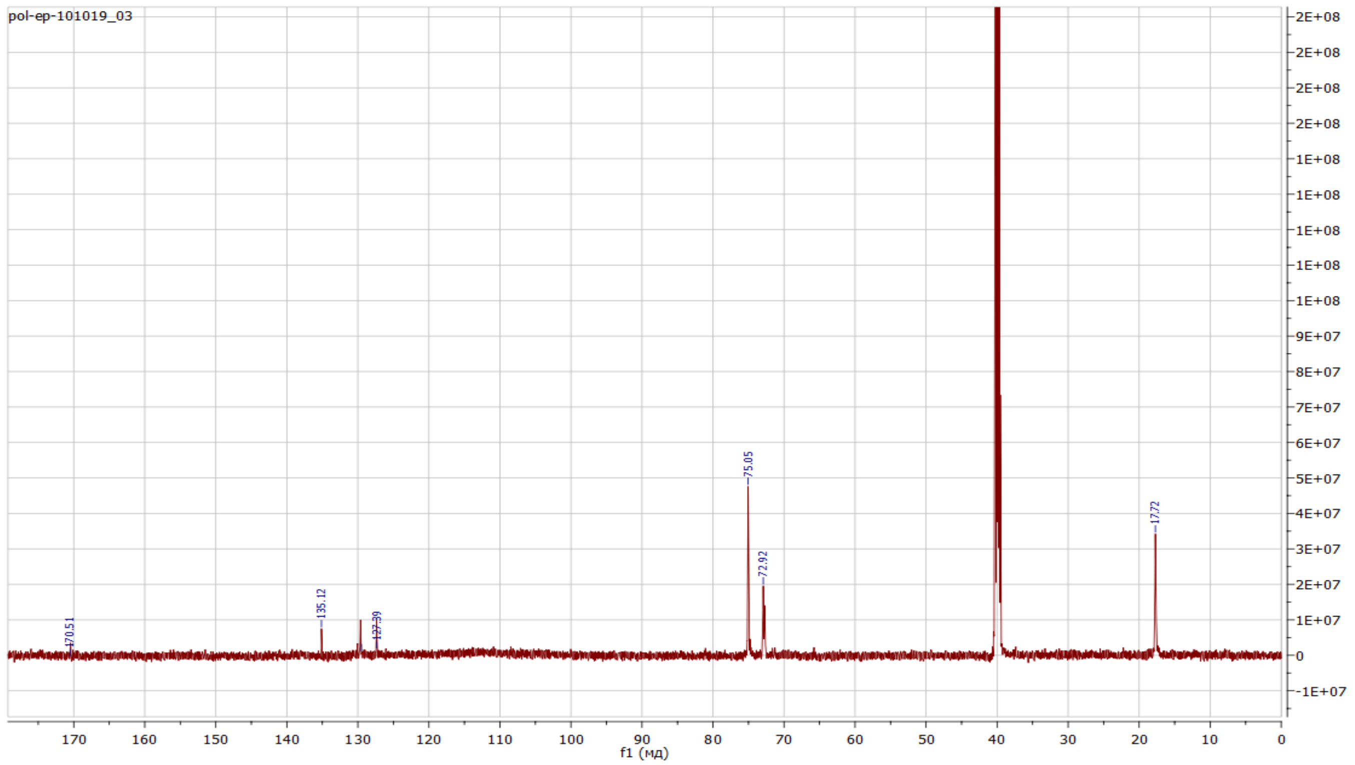

Figure S8. ^{13}C NMR spectrum of PU-V3.0 in DMSO-d_6.

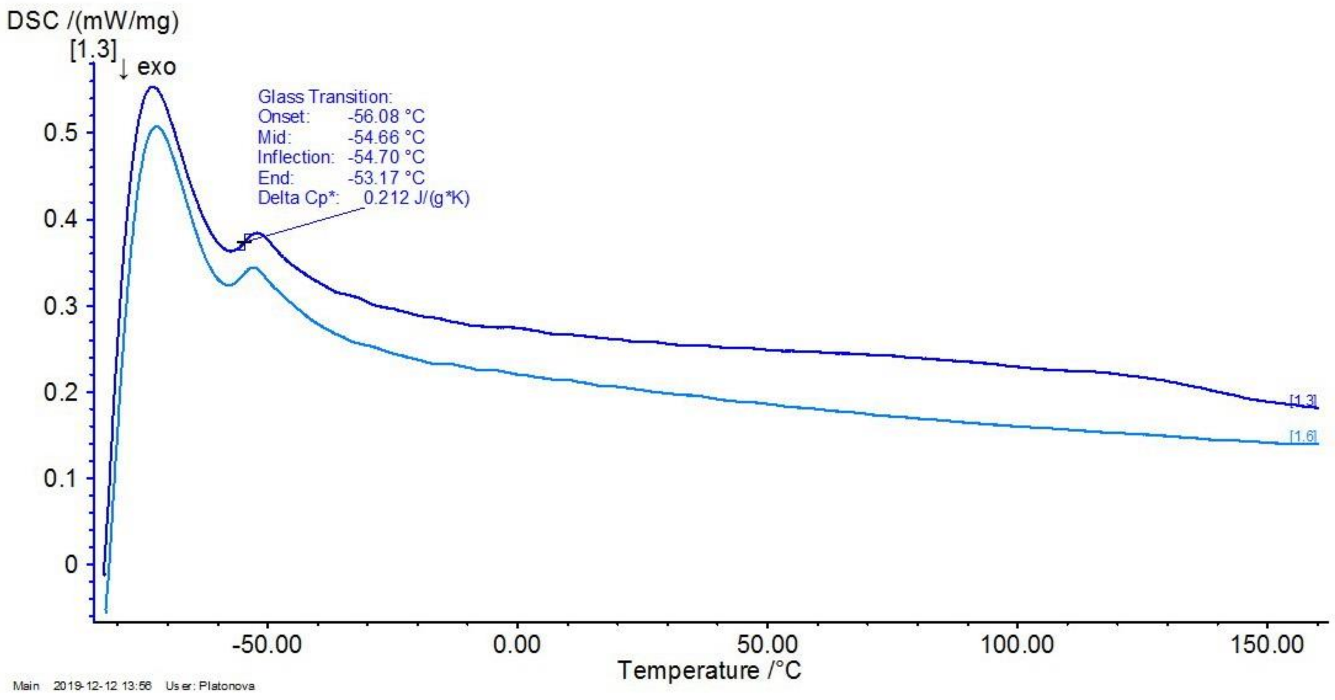

Figure S9. DSC curves for PU-V1.0 at 5 K/min.

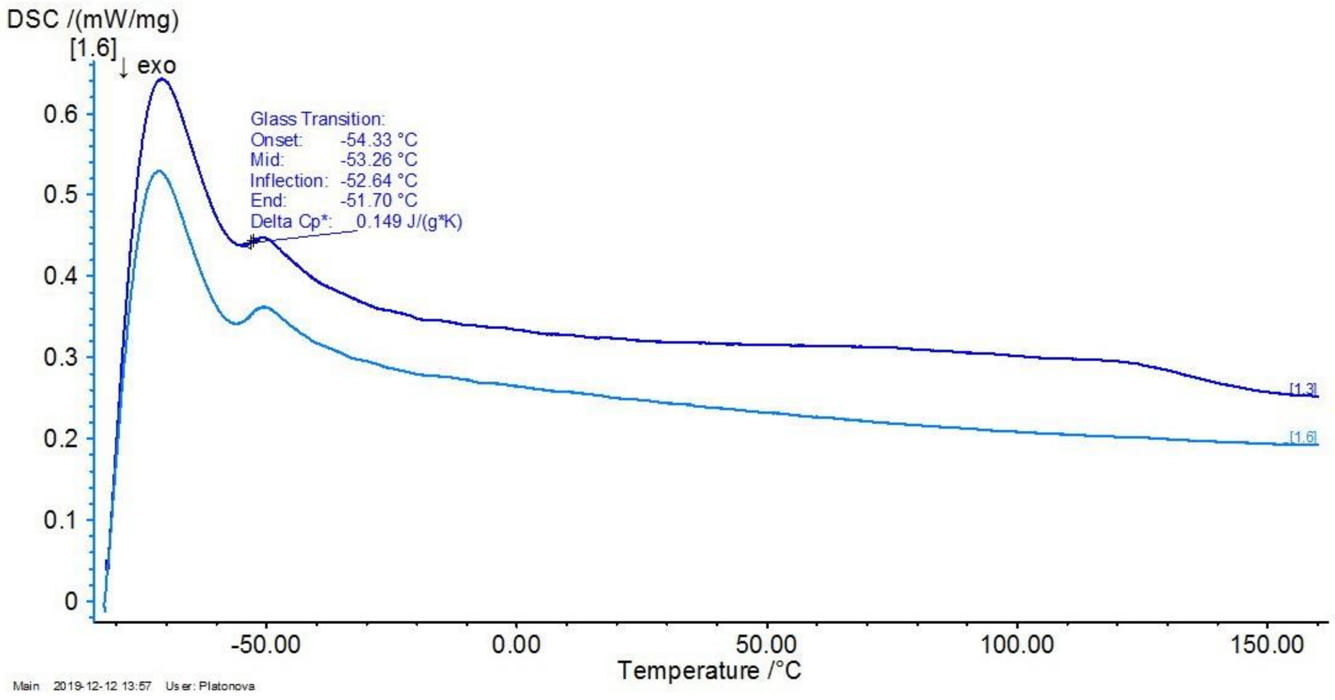

Figure S10. DSC curves for PU-V2.0 at 5 K/min.

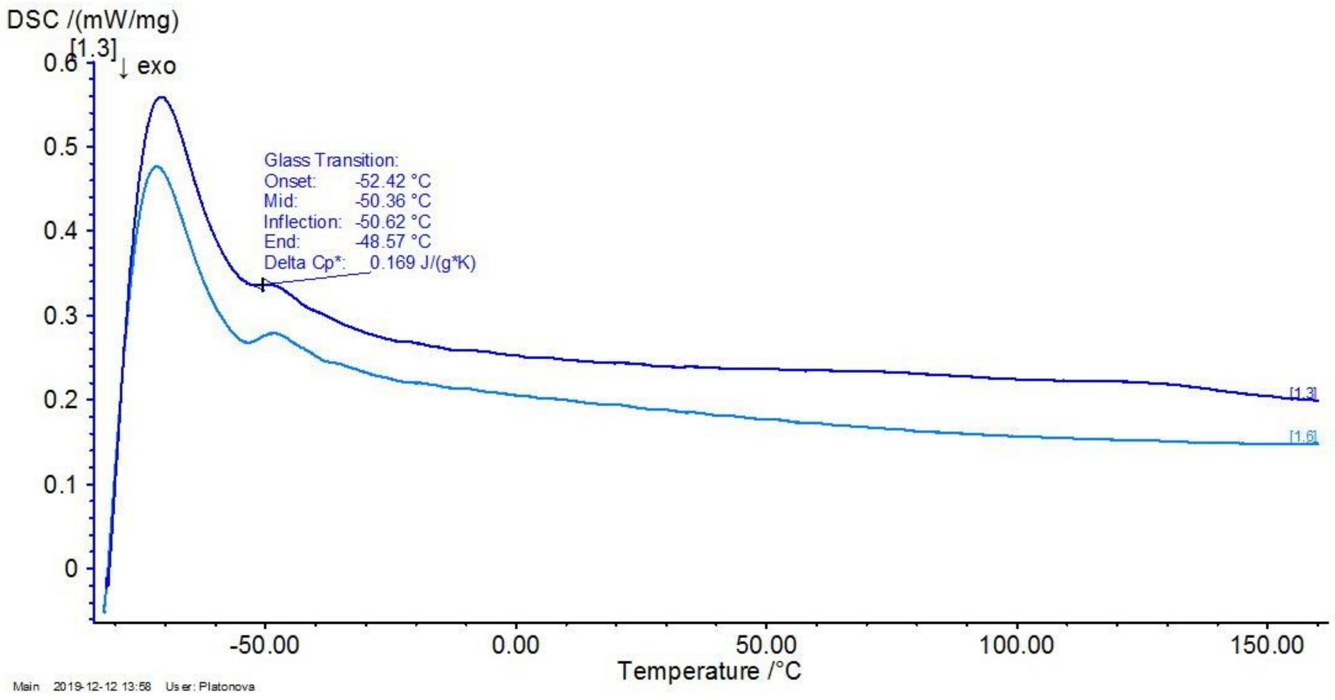

Figure S11. DSC curves for PU-V3.0 at 5 K/min.

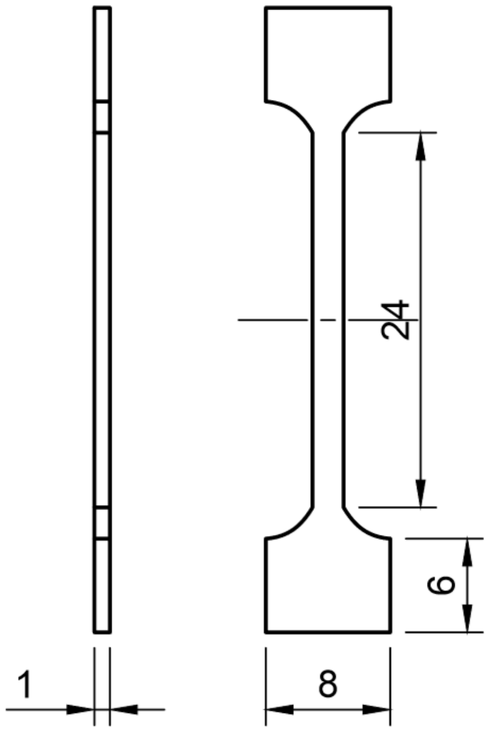

Figure S12. Shape of tensile strength samples of PU-L1.X-3.X (X =0-5).

Tensile tests were performed with Zwick Roell Z100. Set of test parameters are shown at Table S1.

Table S1. Tensile test parameters.

Preload, N	1.2
Preload speed, mm/min	50
Test load speed, mm/min	150/800

Value of preload was chosen in accordance with accuracy of the force sensor (± 1 N) for getting rid of compressive stresses in material, which occur after installation of test samples in the clamps of tensile-machine).

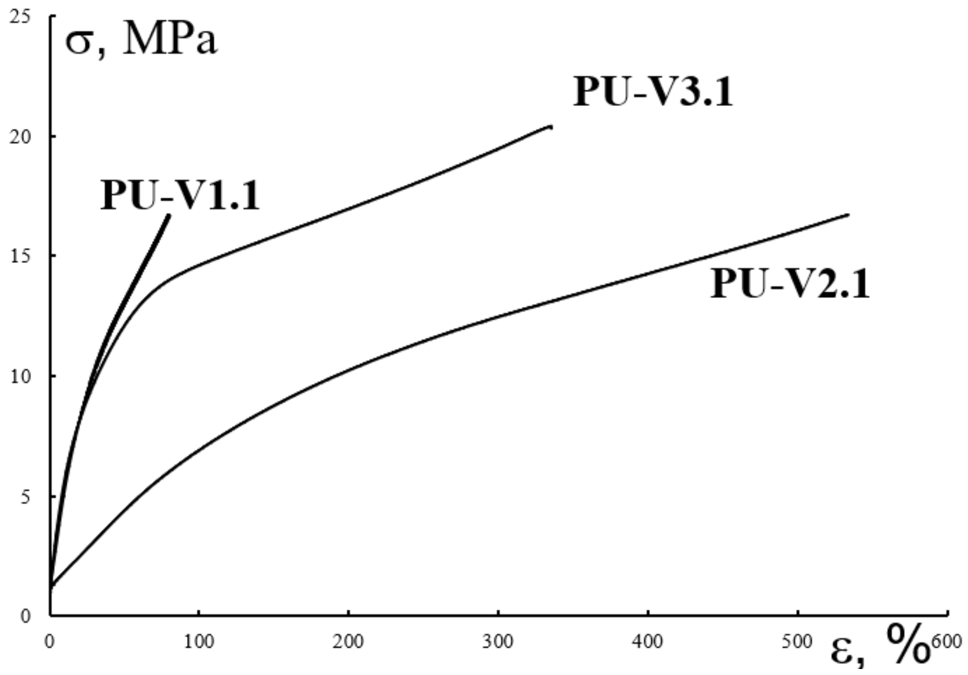

Figure S13. Loading diagrams for samples PU-V1.1-3.1.

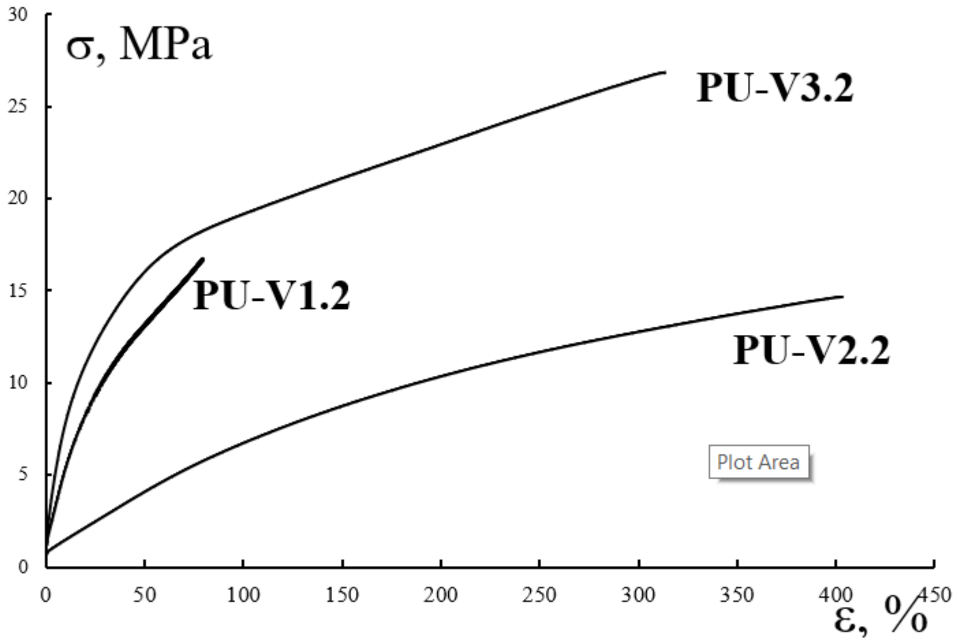

Figure S14. Loading diagrams for samples PU-V1.2-3.2.

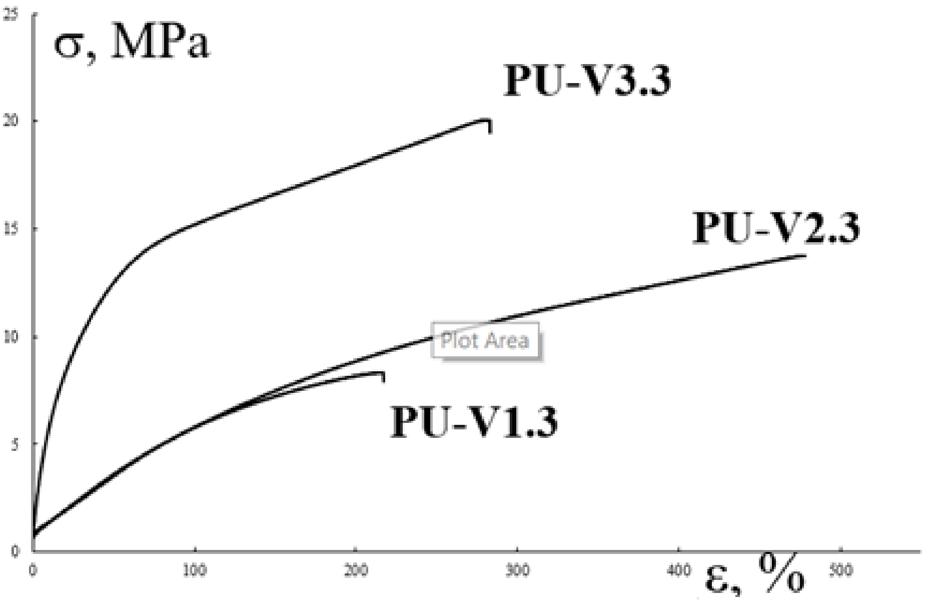

Figure S15. Loading diagrams for samples PU-V1.3-3.3.

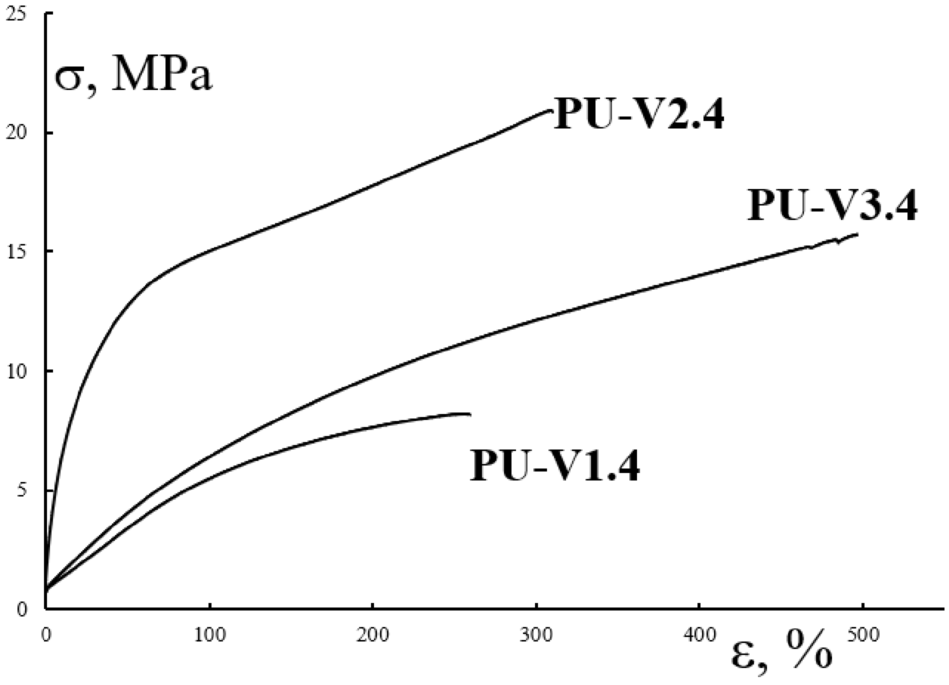

Figure S16. Loading diagrams for samples PU-V1.4-3.4.

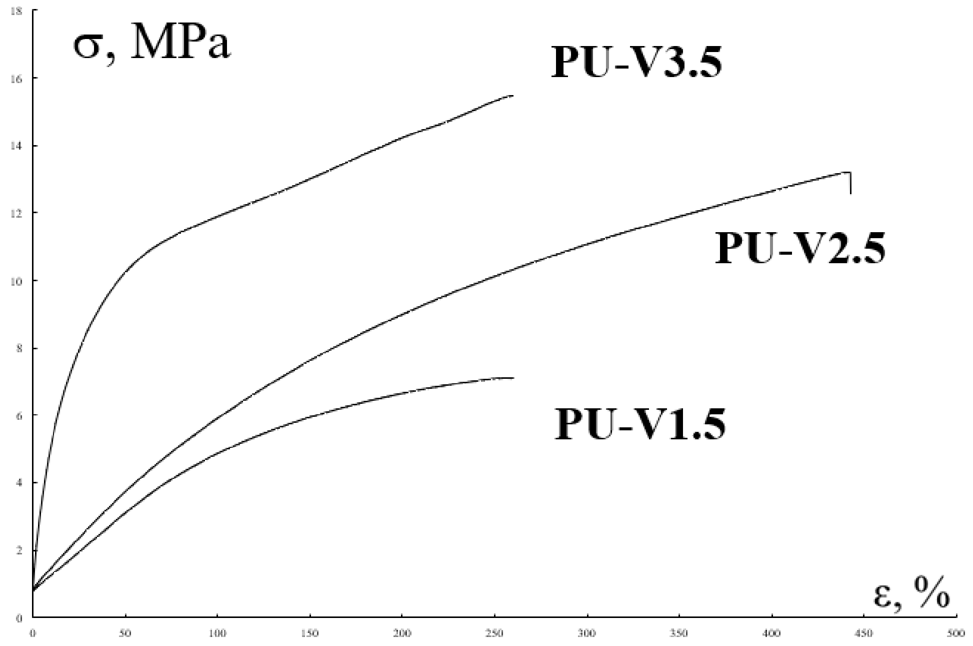

Figure S17. Loading diagrams for samples PU-V1.5-3.5
